# Potential Association of the Oral Microbiome with Trimethylamine N-Oxide Quantification in Mexican Patients with Myocardial Infarction

**DOI:** 10.1155/2024/3985731

**Published:** 2024-02-20

**Authors:** Paulina Hernández-Ruiz, Alma R. Escalona Montaño, Luis M. Amezcua-Guerra, Héctor González-Pacheco, Elena Niccolai, Amedeo Amedei, María M. Aguirre-García

**Affiliations:** ^1^Unidad de Investigación UNAM-INC, División de Investigación, Facultad de Medicina, Universidad Nacional Autónoma de México, Instituto Nacional de Cardiología Ignacio Chávez, Ciudad de México, Mexico; ^2^Departamento de Inmunología, Instituto Nacional de Cardiología Ignacio Chávez, Ciudad de México, Mexico; ^3^Unidad de Cuidados Coronarios, Instituto Nacional de Cardiología Ignacio Chávez, Ciudad de México, Mexico; ^4^Department of Experimental and Clinical Medicine, University of Florence, Florence 50134, Italy; ^5^Interdisciplinary Internal Medicine Unit, Careggi University Hospital, Florence 50134, Italy

## Abstract

Many attempts have been proposed to evaluate the linkage between the oral–gut–liver axis and the mechanisms related to the diseases' establishment. One of them is the oral microbiota translocation into the bloodstream, liver, and gut, promoting a host dysbiosis and triggering the presence of some metabolites such as trimethylamine N-oxide (TMAO), known as a risk marker for cardiovascular disease, and especially the myocardial infarction (MI). In the present pilot study, the involvement of oral dysbiosis related to the presence of TMAO has been considered an independent component of the standard risk factors (SRs) in the development of MI, which has not been previously described in human cohorts. A positive and significant correlation of TMAO levels with *Porphyromonas* was identified; likewise, the increase of the genus *Peptidiphaga* in patients without SRs was observed. We determined that the presence of SRs does not influence the TMAO concentration in these patients. This report is the first study where the relationship between oral dysbiosis and TMAO is specified in the Mexican population. Our findings provide information on the possible contribution of the oral pathogens associated with gut dysbiosis in the development of MI, although further analysis should be performed.

## 1. Introduction

Acute myocardial infarction (MI) represents a leading cause of mortality worldwide [1] and in the Mexican population [2], which leads to the analysis of the different conditions related to the development of this disease. Recently, it has been described the influence of standard modifiable risk factors in the mortality rate of MI patients, which include hypertension, hypercholesterolemia, diabetes, and smoking, and those patients without these traditional factors [3]; the latter represents the highest mortality rate associated with an ST-segment elevation MI (STEMI) [4]. As well, there have been considered other independent modifiable risk factors, such as psychosocial factors, alcohol intake, physical inactivity, and diet [5].

Likewise, a linkage between the diet and the gut microbiota (GM) has been reported, triggering the presence of some GM metabolites, such as trimethylamine N-oxide (TMAO), associated with high cardiovascular risk, the development of a major adverse cardiac event (MACE), and a high mortality rate [6]

Trimethylamine is produced by the GM from the diet-nutrient metabolism of carnitine, choline, and phosphatidylcholine; then, its oxidation by the hepatic flavin monooxygenase produces the TMAO, which is related to a platelet hiper-reactivity, and the accumulation of foam macrophages promoting atherosclerosis [6–8].

According to the GM analysis in STEMI patients, other cohorts have documented a higher abundance in the phyla *Proteobacteria* compared to healthy controls (HCs) [9], while STEMI patients with hyperglycemia present a higher abundance of *Bacteroidetes* [10]. These findings are related to gut dysbiosis. Further, in STEMI patients, a positive correlation has been described between the plasmatic TMAO levels and some GM taxa, such as *Aerococcaceae* [8].

*Prevotella* has been linked to high levels of TMAO in STEMI patients with hyperglycemia, according to an analysis of the microbiota in thrombi. Interestingly, the abundance of this genus is linked to a lower survival range in a 1-year follow-up of these patients [10]. Similarly, in an analysis carried out in STEMI patients' thrombus biopsies, species of the gut and oral microbiota were identified [9] and also found in the bloodstream, suggesting an alteration of gut barrier permeability due to intestinal hypo-perfusion related to the ischemia produced by MI [11].

According to some research, oral and gut dysbiosis may be related. In STEMI patients from an Asian population, the presence of oral and gut dysbiosis was correlated with the thrombus biopsy microbiome; the latter presented a greater similarity with the diversity of the oral microbiome compared to the intestinal niche [9]. Also, there is a linkage between poor oral hygiene and gut dysbiosis, which has been identified by a greater abundance of *Bacteroidetes* [12]. The same linkage was documented in patients with severe periodontitis, where the presence of saliva-derived species was described in the GM analysis with a higher proportion compared to healthy subjects [13]. Finally, the participation of oral cavity pathogens such as *Porphyromonas gingivalis* in intestinal metabolism has been analyzed in murine models, identifying a significant increase in plasmatic concentration in models of induced periodontitis; however, this mechanism has not been detailed in humans [14].

Given the importance of TMAO and oral dysbiosis as an independent component of the standard risk factors (SRs) in the development of MI, the aim of this study was to identify a relationship between the oral microbiota and TMAO, metabolite related to the gut dysbiosis in STEMI patients with the presence of SRs and without them.

## 2. Materials and Methods

### 2.1. Study Patients

This is an observational and cross-sectional study, where we enrolled patients with a first STEMI event of fewer than 72 hr of evolution upon admission to the Coronary Care Unit of the National Institute of Cardiology in Mexico City, Mexico (Instituto Nacional de Cardiología “Ignacio Chávez”). The STEMI diagnosis was established by analyzing myocardial markers and the electrocardiogram findings [15]. We evaluated patients with the presence of SRs, such as hypertension, diabetes, dyslipidemia, and current smoking, or patients with the absence of these risk factors [3].

### 2.2. Biological Samples

Upon patient admission, blood samples were collected in polyethylene terephthalate tubes (BD Vacutainer) with EDTA for plasma, then obtained by centrifugation and immediately stored at −80°C for TMAO quantification. For the DNA extraction, 24 hr upon patient's admission, we collected the supragingival dental plaque samples of all dental organs' vestibular and lingual surfaces with a sterilized 3/4 Gracey curette and transferred them to a polypropylene sterile container (Eppendorf tube) with 70% ethanol and stored at −20°C [16].

### 2.3. TMAO Quantification

TMAO was quantified in plasma samples by ultra-performance liquid chromatography–tandem mass spectrometry (UPLC–MS/MS) and analyzed in an AB SCIEX API 4000 spectrometer. For the standard preparation, TMAO was dissolved in 1.0 N HCl to achieve 1 mg/mL as stocking solutions. The standard was diluted in pure water (with 0.1% formic acid) to get gradient concentrations from 0.2 to 50 ng/mL, respectively. For the sample preparation, 100 *μ*L of the sample was mixed with 300 *μ*L of ice-cold methanol in a 2 mL tube and vortexed for 1 min. The mixture was centrifuged at 12,000 rpm and 4°C for 10 min. The supernatant was transferred to the other tube and filtered through a 0.22 *μ*m membrane filter. The sample was further diluted in pure water (with 0.1% formic acid) with 10 and 100 folds. About 10 *μ*L of the sample was injected into the UPLC–MS/MS for the analysis, which was performed by an external laboratory following their own validated procedure (Creative Proteomics (USA, https://www.creative-proteomics.com)). In the performed analysis, the coefficient of variation (CV%) reported was 2.3 for four samples; in addition, we provided the TMAO analyte product information (cat. 1184-78-7, B412905, TRC, Canada).

### 2.4. Metagenomic Analysis of Oral Microbiota

We performed the analysis of the oral microbiota diversity by 16S rRNA sequencing (V3–V4 region); the taxonomy was assigned using the Human Oral Microbiome (eHOMD, v.15.2) pretrained for the V3–V4 region [17]. The DNA extraction and the 16s rRNA sequencing methods were previously described by our group [16]. The alpha-diversity analysis was performed using the Shannon metric, while beta-diversity was estimated using the Bray–Curtis dissimilarity index and its visualization using principal coordinate analysis. The differences between groups were performed by permutational multivariate analysis of variance (PERMANOVA, adonis, R, 999 permutations). The differential taxonomic analysis was performed by DESeq2 (v.1.42.0, R package) [18].

### 2.5. Statistical Analysis

The statistical analysis for noncontinuous variables was performed using the Fisher exact test and Mann–Whitney for the continuous variables; the calculation of the quartile range was performed using Tukey's Hinges. The *p*  ≤ 0.05 was set for significance for all the analyses. The R packages “ampvis2′ (v.2.7.23), “corrplot” (v.0.92), and “ggplot2′ (v.3.3.6) were used for graphic representation. Spearman correlation coefficients were computed to assess the association between variables. *p*-Values were not corrected for multiple comparisons since it is an exploratory analysis.

## 3. Results

### 3.1. Patients' Clinical Assessment

Sixteen patients who developed the STEMI, were enrolled in the study, and their clinical and demographical characteristics are reported in Table 1.

Patients were divided into two groups according to the identification of one or more SRs and unidentified factors (Non-SR). The enrolled patients ranged in age from 36 to 78, and we only enrolled one female patient. The two groups did not differ significantly in terms of age and sex.

The prevalence of subjects presenting an affection of the descending anterior artery was higher in the SR group (*p*=0.036, Fisher), which presented a lower atherogenic index compared to the Non-SR group (*p*=0.010, Fisher). We also found a borderline significantly different expression of cardiac troponin I and low-density lipoprotein (*p*=0.051) between groups.

### 3.2. Oral Microbiota Composition

**Figure 1:**
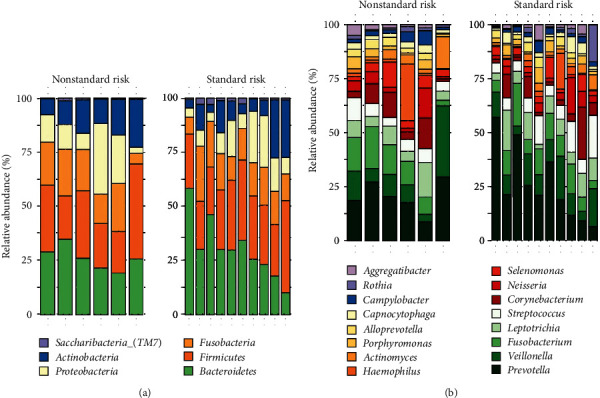
Bar plot with the relative abundance of phyla (a) and genera (b) of the oral microbiota of patients without and with associated risk factors.

Regarding the taxonomic composition, the most abundant taxa (phyla and genera) observed in the patients' group are reported in Figure 1. In general, *Bacteroidetes* was the most abundant phylum, with higher abundance in the standard risk group (30.5% against 26.2%) (Figure 1(a)). At the genus level, *Prevotella* was the most abundant genus, showing to be more abundant in the SR group (21.5% against 17.5%) (Figure 1(b)).

### 3.3. TMAO Plasmatic Levels and Oral Genera

**Figure 2:**
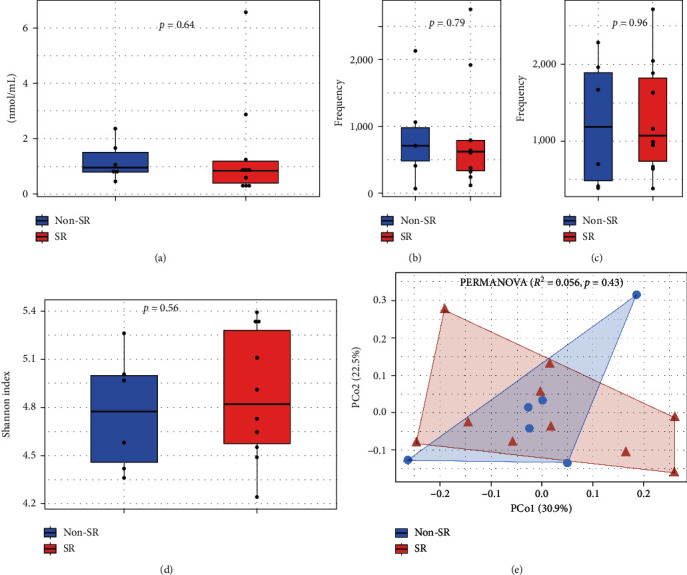
Relation of oral microbiota and trimethylamine N-oxide (TMAO) quantification in myocardial infarction patients according to cardiovascular risk factor group. (a) Trimethylamine N-oxide quantification by risk factor group (nmol/mL. *p* value, Mann–Whitney). Frequency of oral genera positively correlated to TMAO quantification by risk factor group, (b) *Aggregatibacter* and (c) *Porphyromonas*. (d) Alpha diversity analysis by Shannon index according to the risk factor group (*p* value, Mann–Whitney). (e) Principal coordinate analysis of beta diversity by Bray–Curtis index related to risk factor group (PERMANOVA, *R*^2^ = 0.056, *p* value=0.43). Blue (patients without standard risk factor “Non-SR”), red (patients with standard risk factor “SR”).

The median concentration of TMAO in STEMI patients was 0.86 nmol/mL (IQR 0.53–1.45) and did not differ significantly among the SR group of 0.84 nmol/mL (IQR 0.33–1.25) and Non-SR group of 0.94 nmol/mL (IQR 0.79–1.66) (Figure 2(a)).

The 16 more abundant genera were shown in, considering the presence of *Porphyromonas* (Figure 2(b)) and *Aggregatibacter* (Figure 2(c)); both genera were included in a correlation analysis below.

We compared the oral microbiota diversity of the two groups of patients. The analysis of alpha diversity (Shannon index) showed nondifference in microbiota biodiversity among the two groups (Figure 2(d)). As shown by the beta diversity analysis, overall, the microbiota composition was similar among groups, with only 9.6% of the variance determined by the presence of one or more risk factors (PERMANOVA *R*^2^ = 0.096, *p*=0.46) (Figure 2(e)).

**Figure 3:**
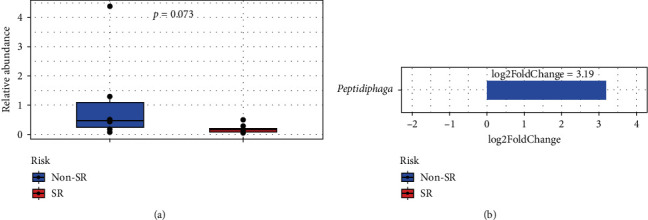
(a) Relative abundance of the genus *Peptidiphaga* and (b) log2FoldChange of this taxa according to cardiovascular risk factor group (*p*.adjust=0.006, log2FoldChange = 3.198428, DESeq2). Blue (patients without standard risk factor “Non-SR”), red (patients with standard risk factor “SR”).

Likewise, a significant difference was observed in the comparative analysis between groups. It should be noted that there is a significant increase in the genus *Peptidiphaga* in the Non-SR group; Figure 3(a) shows the relative abundance of the named genus, while the comparative analysis between groups is represented in Figure 3(b) (*p*.adjust = 0.006, log2FoldChange = 3.19, DESeq2).

### 3.4. Correlation Analysis

The potential correlation between the clinical parameters, TMAO levels, and the oral microbial taxa was assessed; also, the abundance of patients' clinical features and oral bacterial taxa was explored (Figures S1 and S2). In Table 2, a moderate positive correlation was found among TMAO levels and with the abundance of *Porphyromonas* genus (Pearson's correlation 0.56, *p*=0.02); the latter genus presented a moderate positive correlation with *Aggregatibacter* (Pearson's correlation 0.73, *p*=0.001). In addition, the TMAO concentration showed a positive correlation with triglycerides (Pearson's correlation 0.51, *p*=0.04) and a negative correlation with high-density lipoprotein (Pearson's correlation −0.51, *p*=0.04).

## 4. Discussion

In this exploratory analysis of TMAO levels in STEMI patients, the range of TMAO concentration was quantified between 0.3 and 6.6 nmol/mL (median 0.86 nmol/mL), which represents a lower range compared to other populations. For instance, STEMI patients from two Asian populations had median values of 5.63 and 2.18 *μ*M (equivalent to nmol/mL), respectively [19, 20]. Similarly, in the work of Gao et al. [8], both STEMI patients and unstable angina patients showed higher median values of plasmatic TMAO (4.35 ± 2.19 and 4.31 ± 2.8 *μ*M). Interestingly, Matsuzawa et al. [19] reported that the TMAO levels even increased significantly in post-STEMI patients over the course of a 10-month period, unrelated to dietary changes or drug treatment. According to the levels of TMAO observed in STEMI patients with different plaque morphologies, a significantly higher TMAO concentration was observed in patients with plaque rupture than in patients with plaque erosion (3.33 *μ*M (2.48–4.57) vs. 1.21 *μ*M (0.86–1.91)); the latter morphology was associated with smaller infarct size and a lower mortality rate. The Killip–Kimball class recorded in our cohort, most patients were diagnosed without clinical signs of heart failure, so we could assume similarities related to the level of TMAO recorded in patients with plaque erosion previously reported [20].

It is relevant to mention that the present analysis did not consider the study of a control group; however, the concentration found in our cohort of patients is lower than the TMAO values reported in two different cohorts of HCs from the Asian population (2.15 ± 0.9 and 1.23 *μ*M (IQR: 0.84–2.42)) [8, 21].

Another point to consider is the comparison across groups, where no significant difference was found, suggesting that the presence of SRs has no effect on TMAO metabolism. In detail, our data are in contrast with the current literature. For instance, a study on Mexican diabetic subjects revealed that they had an increase of 48.2% compared to healthy subjects [22]. In another report, Sheng et al. [21] reported significantly higher TMAO levels in the presence of multivessel disease, but we did not find a different expression in our patients with ≥2 affected blood vessels. Finally, patients with MACE related to the presence of SRs, such as hypertension, diabetes, or a previous MI, showed increased levels of TMAO (median of 5.0 *μ*M) [6], while our cohort showed TMAO levels (<1 nmol/mL).

In general, we observed a higher abundance of *Prevotella*, this genus had been related to a cardiovascular risk increase [9]. In the differential analysis, we reported a significant increase in the genus *Peptidiphaga* in the Non-SR group; in this case, the literature associated the presence of this genus with the samples taken from the posterior teeth [23]. Also, *Peptidiphaga* in oral microbiota was related to a vegetarian diet and anti-inflammatory profile, supporting the assumption related to changes in oral cavity diversity related to the dietary and anti-inflammatory profile, although this finding has to be correlated with the patient´s dietary pattern [24].

There is evidence of the participation of *Olsenella uli* in the TMAO metabolism [25]; this microorganism had been described in oral and respiratory infections and presented in GM [26, 27], although we did not find a correlation in the present analysis. Instead, we found a positive correlation between the TMAO concentration and the oral genus *Porphyromonas*. The genus *Porphyromonas* is related to the development of periodontal disease, persistent local inflammation, as well as rheumatoid arthritis [28], and importantly, they have been identified in patients with acute coronary syndrome [29]. Moreover, there is evidence that *P. gingivalis* virulence factors, such as LPS and gingipains, degrade the intercellular junction proteins, affecting the permeability of the blood–brain barrier, related to neurological disorders [30]. Also, we found a strong positive correlation between the genera *Porphyromonas* and *Aggregatibacter*; this finding resembles a previous report, showing that *Aggregatibacter actinomycetemcomitans* contributes to the reduction of H_2_O_2_ produced by *Streptococcus sanguis*, and consequently, promotes an increase in the abundance of *P. gingivalis* [31].

There are reports that established the particular association between periodontal pathogens on liver alterations. First, the administration of *P. gingivalis* in murine models with induced periodontitis has been related to elevated levels of TMAO [14], while in relation to *A. actinomycetemcomitans*, an association has been reported in patients with nonalcoholic fatty liver disease (NAFLD), where a role of this species in the alteration of hepatic metabolism has been demonstrated in murine models [32]. Also, the influence of *P. gingivalis* in patients with NAFLD, considering an alteration in the liver promoted by this pathogen [33, 34].

As previously indicated, there is a connection between the oral-GM. In fact, there are studies describing the relationship between anaerobic periodontal pathogens with gut dysbiosis in healthy patients [12], as well as in subjects diagnosed with ulcerative colitis [35]. Likewise, Kwun et al. [9] showed a higher proportion of *P. gingivalis* in the oral cavity of STEMI patients, related to a state of intestinal dysbiosis, identified by a greater abundance of *Proteobacteria* and a lower abundance of short-chain fatty acid-producing bacteria; the decreasing of these metabolites is related to an alteration of the gut barrier permeability.

## 5. Conclusions

Based on the findings, we found the relationship of the *Porphyromonas* genus with the TMAO levels in patients with STEMI, suggesting the participation of this oral pathogen in the liver–gut dysbiosis as described in previous reports, determined by the TMAO quantification in these patients. Moreover, it was possible to determine that the presence or absence of SRs does not influence the concentration of TMAO in these STEMI patients, although the increase of the genus *Peptidiphaga* in Non-SR patients suggests a change in the oral diversity between groups. This exploratory study was the first to describe a linkage between oral dysbiosis and the TMAO concentration in Mexican STEMI patients; indeed, further studies with a sizable cohort will be necessary to consider the effects of different variables such as age and gender.

## Figures and Tables

**Table 1:** Summary of cohort characteristics.

	Total (*N* = 16)	Without risk factor (*N* = 6)	Risk factor (*N* = 10)	*p* Value
Trimethylamine N-oxide (nmol/mL)^a^	0.86 (0.53–1.45)	0.94 (0.79–1.66)	0.84 (0.33–1.25)	0.588
Age (years)	61 (36–78)	67 (48–78)	59 (36–70)	0.302
Men, *n* (%)	15 (93.7)	5/6	10/10	0.375
Patient medical history				
Diabetes, *n* (%)	5 (31.2)	Non	5 (50)	
Hypertension, *n* (%)	7 (43.7)	Non	7 (70)	
Dyslipidemia, *n* (%)	3 (18.7)	Non	3 (30)	
Current smoking, *n* (%)	4 (25)	Non	4 (40)	
Clinical features				
Killip–Kimball > 2	3 (18.7)	Non	3 (30)	0.25
MACE, *n* (%)	4 (25)	Non	4 (40)	0.234
TIMI ≥ 2*n* (%)	10 (62.5)	3 (50)	7 (70)	0.607
Affected blood vessels ≥ 2*n* (%)	11 (68.7)	3 (50)	8 (80)	0.299
Anterior descending, *n* (%)	13 (81.2)	3 (50)	10 (100)	0.036^*∗*^
Circumflex, *n* (%)	8 (50)	3 (50)	5 (50)	1.000
Right coronary, *n* (%)	11 (68.7)	4 (66.7)	7 (70)	1.000
Body mass index^a^	26.9 (25.6–28.7)	28.6 (27.0–32.9)	26.5 (25.2–27.8)	0.221
Biochemical markers				
cTnI (ng/mL)^a^	32.9 (4.25–260.9)	441.8 (23.7–4101.0)	11.8 (2.3–69.6)	0.051^*∗*^
Total cholesterol (mg/dL)^a^	164.1 (133.3–175.3)	179.3 (159.5–232.3)	152.4 (131.0–165.0)	0.104
HDL (mg/dL)^a^	35.5 (28.5–43.3)	31.5 (31.4–38.3)	35.5 (28.2–50.2)	0.664
LDL (mg/dL)^a^	94.9 (88.0–124.9)	137.2 (112.5–171.1)	91.9 (86.4–95.6)	0.051^*∗*^
Triglycerides (mg/dL)^a^	138.5 (112.7–170.8)	131.5 (112.7–170.3)	143.1 (97.1–182.0)	0.958
Atherogenic index^a^	3.05 (2.4–4.3)	4.5 (3.4–4.89)	2.6 (1.9–3.09)	0.008^*∗*^
Glucose (mg/dL)	127 (115.5–157.5)	125 (116.0–136.0)	129 (115.0–212.0)	0.786

^a^Median (IQR), *p* value Mann–Whitney or Fisher's exact test. MACE: major adverse cardiovascular event, TIMI: thrombolysis in myocardial infarction score. cTnI: cardiac troponin I, HDL: high-density protein, LDL: low-density protein.  ^*∗*^*p* < 0.05.

**Table 2:** Correlation test of trimethylamine N-oxide (TMAO) quantification, oral microbiota diversity, and biochemical parameters in STEMI patients.

	Correlation	*p* Value	95% CI	
			Lower	Upper
TMAO (log)				
* Porphyromonas*	0.56	0.02	0.071	0.834
Triglycerides	0.51	0.04	0.004	0.813
High-density lipoprotein	−0.52	0.04	−0.816	−0.148
*Porphyromonas*				
* Aggregatibacter*	0.73	0.001	0.383	0.903

Pearson's correlation. CI: confidence interval.

## Data Availability

The raw 16S sequences have been deposited at the National Center for Biotechnology Information (NCBI)-Sequence Read Archive (SRA) under project PRJNA878487.

## Supplementary Materials

Supplementary Materials Figure S1: correlation matrix of oral microbiota diversity and trimethylamine N-oxide (TMAO) 2 quantification in myocardial infarction patients. Figure S2: correlation matrix of trimethylamine N-oxide (TMAO) quantification with 7 biochemical and clinical parameters in myocardial infarction patients.
