# Supplementary material for: Potential Association of the Oral Microbiome with Trimethylamine N-Oxide Quantification in Mexican Patients with Myocardial Infarction
Source: Mediators Inflamm. 2024 Feb 20;2024:3985731. doi: 10.1155/2024/3985731 (PMC10898950; doi:10.1155/2024/3985731)
Supplement: Supplementary Materials — Figure S1: correlation matrix of oral microbiota diversity and trimethylamine N-oxide (TMAO) 2 quantification in myocardial infarction patients. Figure S2: correlation matrix of trimethylamine N-oxide (TMAO) quantification with 7 biochemical and clinical parameters in myocardial infarction patients. [file 3985731.f1.pdf]

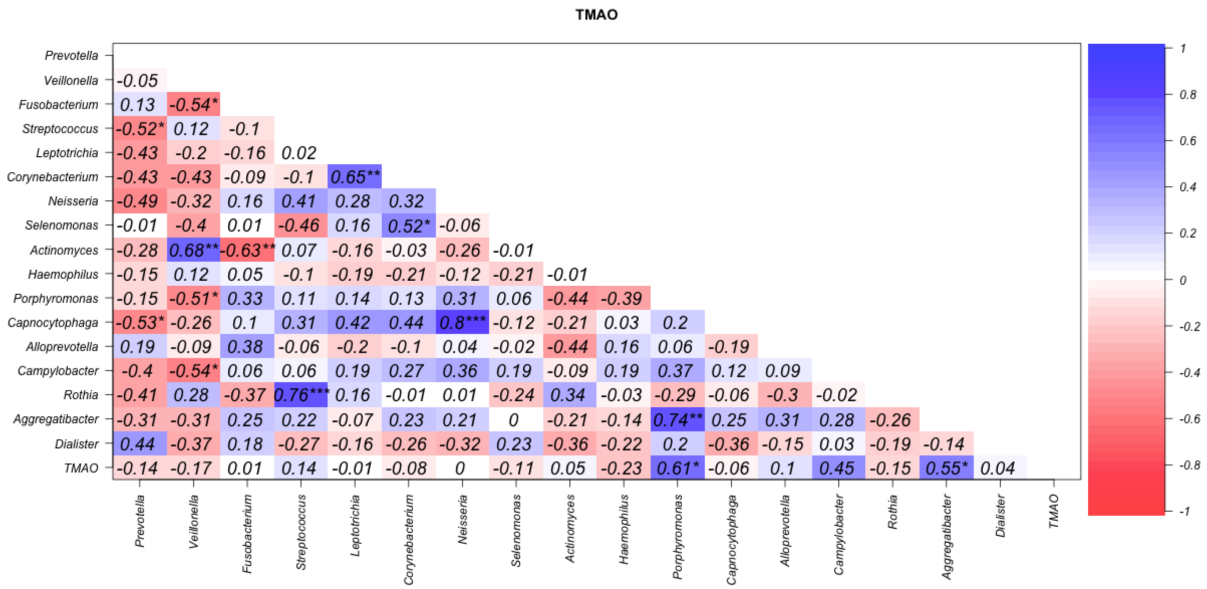

Supplementary figure 1. Correlation matrix of oral microbiota diversity and trimethylamine N-oxide (TMAO) quantification in myocardial infarction patients. p value (*<0.05, ** <0.01, ***<0.001).

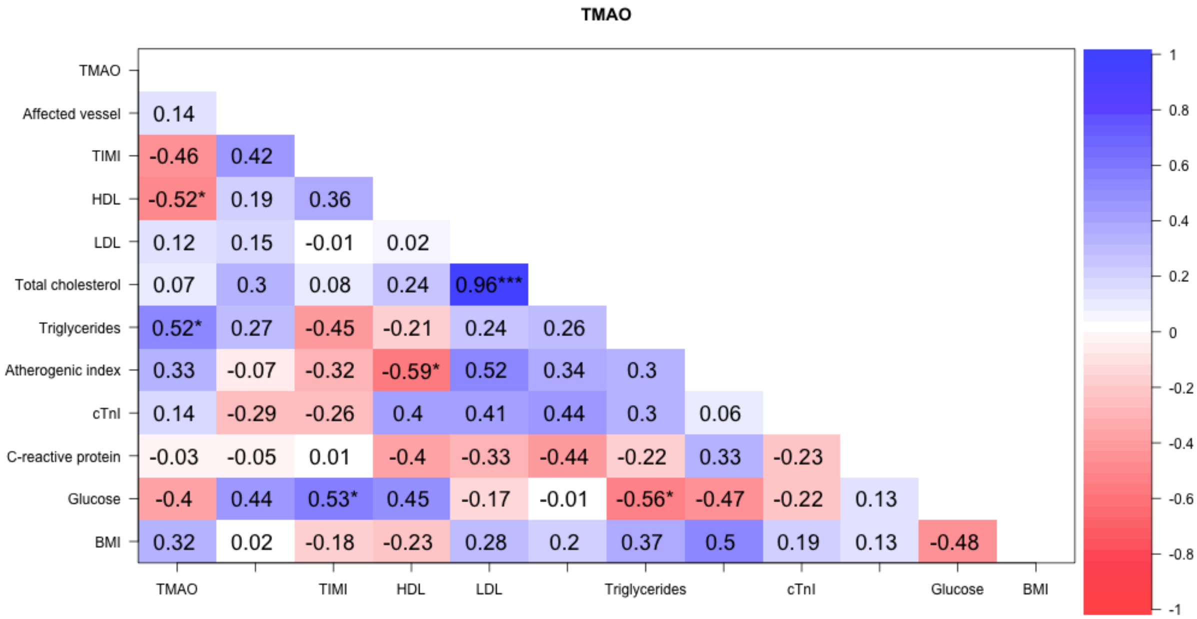

Supplementary figure 2. Correlation matrix of trimethylamine N-oxide (TMAO) quantification with biochemical and clinical parameters in myocardial infarction patients. p value (*<0.05, ** <0.01, ***<0.001). TIMI: Thrombolysis in Myocardial Infarction score, HDL: high density protein, LDL: low density protein, cTnI: Cardiac troponin I, BMI: body mass index.
